# Supplementary material for: Selective sweeps on different pigmentation genes mediate convergent evolution of island melanism in two incipient bird species
Source: PLoS Genet. 2022 Nov 1;18(11):e1010474. doi: 10.1371/journal.pgen.1010474 (PMC9624418; doi:10.1371/journal.pgen.1010474)
Supplement: S1 Table — (DOCX) [file pgen.1010474.s001.docx]

Table S1: Genes within the association peaks.

| **Comparison** | **Contig** | **Chromosome** | **Number of annotations** | **Genes** |
| --- | --- | --- | --- | --- |
| SASC vs. Makira | 400 | 11 | 15 | *TANGO6, GAS8, DBNDD1, DEF8, TUBB3, MC1R, TCF25, SPIRE2, FANCA, ZNF276, VPS9D1, SPATA2L, CDK10, LOC100232461, LOC115496849* |
| Ugi vs. Makira | 1042 | Z | 1 | *LOC115491070* |
| Ugi vs. Makira | 224 | Z | 15 | *SLC44A1, SLC27A6, ISOC1, ADAMTS19, MINAR2, CHSY3, HINT1, LYRM7, CDC42SE2, SPTLC1, ROR2, NFIL3, DIRAS2, GADD45G, LOC116806816* |
| Ugi vs. Makira | 5 | 1 | 23 | *EFHC2, FUNDC1, DIPK2B, MIR221, ICOSLG, GATD3A, PWP2, TRAPPC10, AGPAT3, PDXK, RRP1B, HSF2BP, SIK1, CRYAA, U2AF1, LOC116808847, LOC115496971, LOC115496975, LOC115496977, LOC115497347, LOC115496981, LOC115496994, LOC115497018* |
| Ugi vs. Makira | 866 | 20 | 10 | *TP53INP2, NCOA6, GGT7, RAB5IF, MYL9, TGIF2, DLGAP4, EPB41L1, SRSF6, L3MBTL1* |
| Ugi vs. Makira | 947 | 20 | 14 | *RALY, EIF2S2, ASIP, AHCY, ITCH, DYNLRB1, FSIP2, CEP250, GDF5, FAM83C, EIF6, MMP24, LOC116806597, LOC116809171* |
| Ugi vs. Makira | 62 | Z | 19 | *PLPP1, MSMP, GALT, SIGMAR1, DCTN3, ENHO, FAM219A, MYORG, NUDT2, UBAP1, DCAF12, NOL6, AQP3, KIAA1328, MAPK6, LOC115491277, LOC105760850, LOC115491042, LOC100226213* |
| Ugi vs. Makira | 318 | 6 | 1 | *LOC116808540* |
