# Supplementary material for: Selective sweeps on different pigmentation genes mediate convergent evolution of island melanism in two incipient bird species
Source: PLoS Genet. 2022 Nov 1;18(11):e1010474. doi: 10.1371/journal.pgen.1010474 (PMC9624418; doi:10.1371/journal.pgen.1010474)
Supplement: S2 Table — (DOCX) [file pgen.1010474.s002.docx]

**Table S2: Details for the samples used in this study.**

| **Subspecies** | **Island** | **Collection date** | **Locality** | **Latitude** | **Longitude** | **Ventral Coloration** | **Sample ID** | **Sex*** |
| --- | --- | --- | --- | --- | --- | --- | --- | --- |
| *megarhynchus* | Makira | 5/16/09 | Kira Kira | -10.5 | 161.9 | Chestnut | MA132 | M |
| *megarhynchus* | Makira | 5/16/09 | Kira Kira | -10.5 | 161.9 | Chestnut | MA133 | M |
| *megarhynchus* | Makira | 5/22/09 | Kira Kira | -10.5 | 161.9 | Intermediate | MA166 | M |
| *megarhynchus* | Makira | 6/21/09 | Star Harbour | -10.8 | 162.2 | Chestnut | MA175 | M |
| *megarhynchus* | Makira | 6/22/09 | Star Harbour | -10.8 | 162.2 | Chestnut | MA180 | M |
| *megarhynchus* | Makira | 6/22/09 | Star Harbour | -10.8 | 162.2 | Intermediate | MA182 | F |
| *megarhynchus* | Makira | 6/23/09 | Star Harbour | -10.8 | 162.2 | Chestnut | MA183 | M |
| *megarhynchus* | Makira | 6/23/09 | Star Harbour | -10.8 | 162.2 | Chestnut | MA184 | M |
| *megarhynchus* | Makira | 6/24/09 | Star Harbour | -10.8 | 162.2 | Intermediate | MA185 | F |
| *megarhynchus* | Makira | 6/24/09 | Star Harbour | -10.8 | 162.2 | Chestnut | MA187 | M |
| *megarhynchus* | Makira | 6/15/11 | Kira Kira | -10.5 | 161.9 | Chestnut | MA193 | F |
| *megarhynchus* | Makira | 3/15/12 | Kira Kira | -10.5 | 161.9 | Chestnut | MA230 | F |
| *megarhynchus* | Makira | 3/15/12 | Kira Kira | -10.5 | 161.9 | Chestnut | MA231 | F |
| *megarhynchus* | Makira | 6/30/12 | Kira Kira | -10.5 | 161.9 | Chestnut | MA250 | F |
| *megarhynchus* | Makira | 3/11/14 | Waimasi | -10.4 | 161.7 | Intermediate | MA434 | M |
| *megarhynchus* | Makira | 3/11/14 | Waimasi | -10.4 | 161.7 | Intermediate | MA435 | F |
| *megarhynchus* | Makira | 3/12/14 | Waimasi | -10.4 | 161.7 | Chestnut | MA440 | M |
| *megarhynchus* | Makira | 3/12/14 | Waimasi | -10.4 | 161.7 | Intermediate | MA441 | F |
| *megarhynchus* | Makira | 6/15/18 | Waimasi | -10.4 | 161.7 | Chestnut | MA704 | M |
| *megarhynchus* | Makira | 6/15/18 | Waimasi | -10.4 | 161.7 | Melanic | MA705 | M |
| *megarhynchus* | Makira | 7/2/18 | Waimasi | -10.4 | 161.7 | Chestnut | MA714 | M |
| *megarhynchus* | Makira | 5/15/09 | Kira Kira | -10.5 | 161.9 | Chestnut | MA129** | M |
| *ugiensis* | Santa Ana | 8/8/06 | Gupuna | -10.8 | 162.5 | Melanic | SA082 | M |
| *ugiensis* | Santa Ana | 8/10/06 | Gupuna | -10.8 | 162.5 | Intermediate | SA085 | M |
| *ugiensis* | Santa Ana | 8/10/06 | Gupuna | -10.8 | 162.5 | Melanic | SA087 | F |
| *ugiensis* | Santa Ana | 8/11/06 | Gupuna | -10.8 | 162.5 | Melanic | SA095 | M |
| *ugiensis* | Santa Ana | 6/17/07 | Gupuna | -10.8 | 162.5 | Melanic | SA105 | M |
| *ugiensis* | Santa Ana | 6/17/07 | Gupuna | -10.8 | 162.5 | Melanic | SA106 | M |
| *ugiensis* | Santa Ana | 6/17/07 | Gupuna | -10.8 | 162.5 | Melanic | SA107 | M |
| *ugiensis* | Santa Ana | 5/7/08 | Gupuna | -10.8 | 162.5 | Melanic | SA121 | M |
| *ugiensis* | Santa Ana | 5/7/08 | Gupuna | -10.8 | 162.5 | Melanic | SA123 | M |
| *ugiensis* | Santa Ana | 5/8/08 | Gupuna | -10.8 | 162.5 | Melanic | SA124 | M |
| *ugiensis* | Santa Ana | 5/7/08 | Gupuna | -10.8 | 162.5 | Melanic | SA125 | F |
| *ugiensis* | Santa Ana | 3/20/13 | Gupuna | -10.8 | 162.5 | Melanic | SA267 | M |
| *ugiensis* | Santa Catalina | 6/22/13 | Santa Catalina | -10.9 | 162.5 | Intermediate | SC275 | F |
| *ugiensis* | Santa Catalina | 6/22/13 | Santa Catalina | -10.9 | 162.5 | Intermediate | SC277 | F |
| *ugiensis* | Santa Catalina | 6/22/13 | Santa Catalina | -10.9 | 162.5 | Intermediate | SC278 | M |
| *ugiensis* | Santa Catalina | 6/22/13 | Santa Catalina | -10.9 | 162.5 | Melanic | SC283 | M |
| *ugiensis* | Santa Catalina | 6/24/13 | Santa Catalina | -10.9 | 162.5 | Melanic | SC296 | M |
| *ugiensis* | Santa Catalina | 6/24/13 | Santa Catalina | -10.9 | 162.5 | Melanic | SC402 | M |
| *ugiensis* | Santa Catalina | 6/24/13 | Santa Catalina | -10.9 | 162.5 | Melanic | SC404 | M |
| *ugiensis* | Ugi | 4/26/08 | Pawa | -10.3 | 161.7 | Melanic | UG108 | M |
| *ugiensis* | Ugi | 4/27/08 | Pawa | -10.3 | 161.7 | Melanic | UG115 | M |
| *ugiensis* | Ugi | 4/27/08 | Pawa | -10.3 | 161.7 | Melanic | UG116 | F |
| *ugiensis* | Ugi | 5/18/09 | Pawa | -10.3 | 161.7 | Melanic | UG147 | M |
| *ugiensis* | Ugi | 5/18/09 | Pawa | -10.3 | 161.7 | Melanic | UG148 | M |
| *ugiensis* | Ugi | 5/19/09 | Pawa | -10.3 | 161.7 | Melanic | UG152 | M |
| *ugiensis* | Ugi | 5/19/09 | Pawa | -10.3 | 161.7 | Melanic | UG155 | M |
| *ugiensis* | Ugi | 5/19/09 | Pawa | -10.3 | 161.7 | Melanic | UG156 | M |
| *ugiensis* | Ugi | 5/20/09 | Pawa | -10.3 | 161.7 | Melanic | UG160 | F |
| *ugiensis* | Ugi | 5/20/09 | Pawa | -10.3 | 161.7 | Melanic | UG161 | M |
| *ugiensis* | Ugi | 6/8/11 | Pawa | -10.3 | 161.7 | Melanic | UG197 | M |
| *ugiensis* | Ugi | 3/13/12 | Pawa | -10.3 | 161.7 | Melanic | UG220 | M |
| *ugiensis* | Ugi | 7/4/13 | Bio | -10.2 | 161.7 | Melanic | UG407 | M |
| *ugiensis* | Ugi | 3/15/14 | Pawa | -10.3 | 161.7 | Melanic | UG448 | M |
| *ugiensis* | Ugi | 3/15/14 | Pawa | -10.3 | 161.7 | Melanic | UG450 | F |
| *ugiensis* | Ugi | 3/15/14 | Pawa | -10.3 | 161.7 | Melanic | UG451 | M |

*We determined sex by calculating the average depth of coverage across all the positions in each of six different contigs. Three of these contigs were autosomal and three were part of the Z chromosome. We subsequently averaged the depth of coverage for the three Z-linked contigs and divided it by the average from the three autosomal contigs. This process produced values around 0.5 for heterogametic females, and values close to 1 for males. Additionally, for a subset of 39 individuals we also determined sex through PCR as described in reference (1). Both methods produced congruent results.

**This sample is labelled as UG129 and not MA129 in the different files from our bioinformatics pipeline (e.g., vcf files), yet was treated correctly as a sample originating from Makira and not from Ugi (as other samples denoted with UG).

**References**

1. N. W. Kahn, J. St. John, T. W. Quinn, Chromosome-specific intron size differences in the avian CHD gene provide an efficient method for sex identification in birds. *The Auk*, 1074–1078 (1998).
