# Supplementary figures and images for: Selective sweeps on different pigmentation genes mediate convergent evolution of island melanism in two incipient bird species
Source: PLoS Genet. 2022 Nov 1;18(11):e1010474. doi: 10.1371/journal.pgen.1010474 (PMC9624418; doi:10.1371/journal.pgen.1010474)

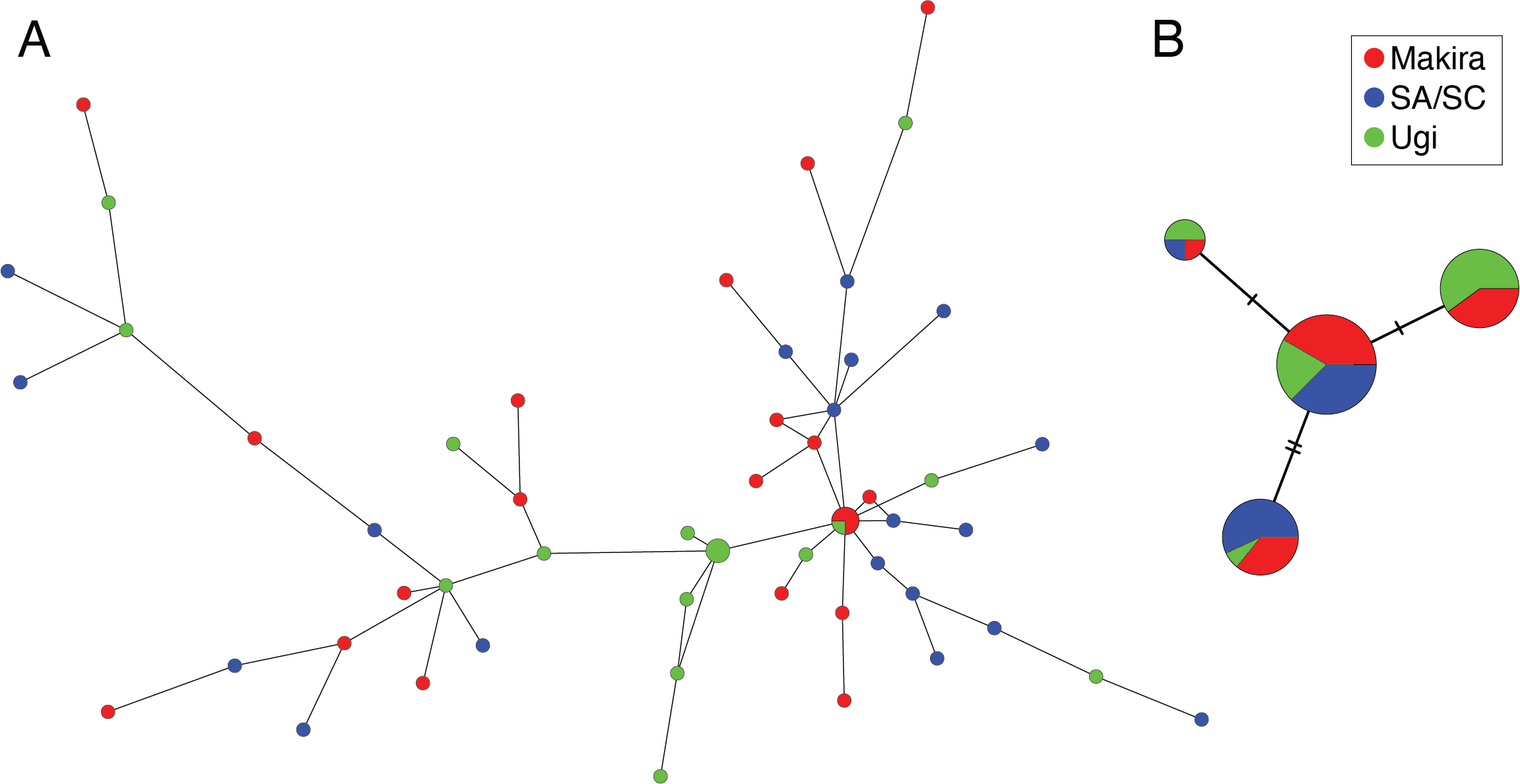

Supplement: S1 Fig — Mitochondrial minimum spanning networks. A. Haplotype network based on a ~17 kbp alignment of the mitochondrial genome. B. Haplotype network based on 650 bp of the mitochondrial COI gene, commonly used for species identification. Branch lengths are proportional to the number of nucleotide differences between haplotypes, which are indicated by short lines on each branch (omitted for simplicity in the case of the full mitochondrial network). (TIF) [file pgen.1010474.s003.tif]

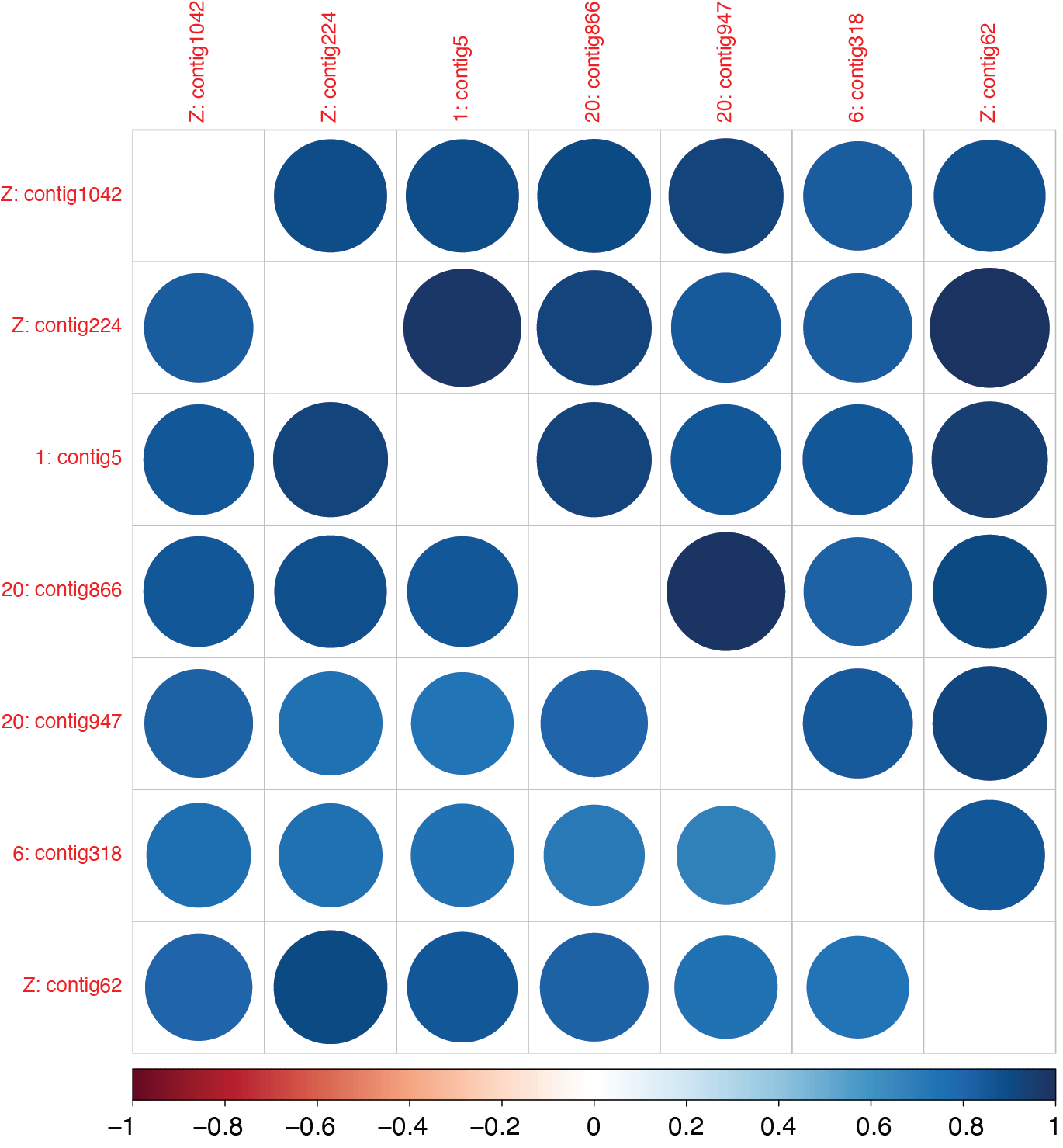

Supplement: S2 Fig — Linkage disequilibrium among association peaks identified in the GWAS conducted with Makira and Ugi individuals. Average (below the diagonal) and maximum (above the diagonal) R2 values among all the statistical outlier sites in the GWAS from different pairs of association peaks. The chromosome and contig to which each peak belongs is indicated in red, and the size and color of the circles denotes the magnitude of LD. (TIF) [file pgen.1010474.s004.tif]

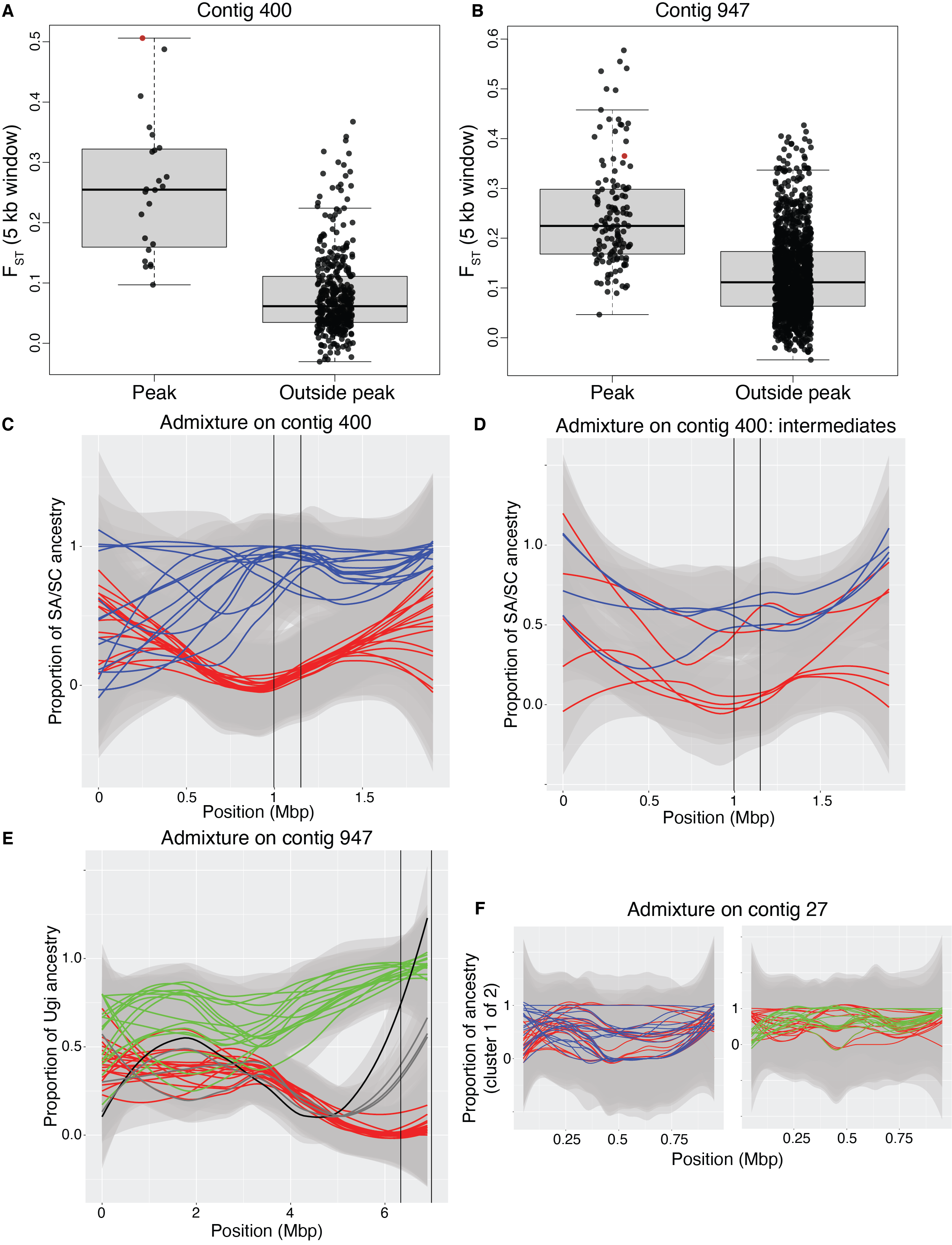

Supplement: S3 Fig — Genetic differentiation within and outside of association peaks. A and B. FST values calculated for 5 kb windows inside and outside association peaks. The plot for contig 400 compares individuals from SA/SC and Makira, while the plot for contig 947 compares individuals from Ugi and Makira. The red dot denotes the window containing the MC1R and ASIP genes. C to F. Smoothed ancestry values across contigs, with association peaks indicated between vertical lines. Ancestry values were calculated in 100 kb sliding windows in Admixture, and the analysis was restricted to Makira vs. SA/SC for contig 400 and Makira vs. Ugi for contig 947. The plot in (C) shows increased separation between individuals from Makira and SA/SC in the peak region on contig 400 and extending approximately 0.5 Mbp in each direction. The plot includes only individuals from Makira and SA/SC that were homozygotes for the Asp119 or Asn119 MC1R mutation, respectively. The plot in (D) shows the six individuals from Makira and the four individuals from SA/SC which were heterozygotes for this mutation and had intermediate plumage, and shows overall more admixture than what is seen in (C). E. Ancestry values across contig 947 showing increased resolution in the peak region and extending approximately 2 Mb downstream. Three individuals from Makira which were heterozygotes for the Tre55 ASIP mutation are labeled in gray (only one of these individuals had intermediate plumage). The single melanic individual from Makira (MA705), which was homozygous for the derived Tre55 mutation, is labelled in black. For all four individuals, Ugi ancestry decreases to levels comparable to other Makira individuals about 1 Mb downstream of the peak. F. Ancestry across contig 27 shows little resolution compared to the association peaks on contigs 400 and 947. We note that ancestry values range from 0 to 1, but that the plots extend beyond this range because of the smoothing algorithm and particularly the uncertainty shown by the [file pgen.1010474.s005.tif]

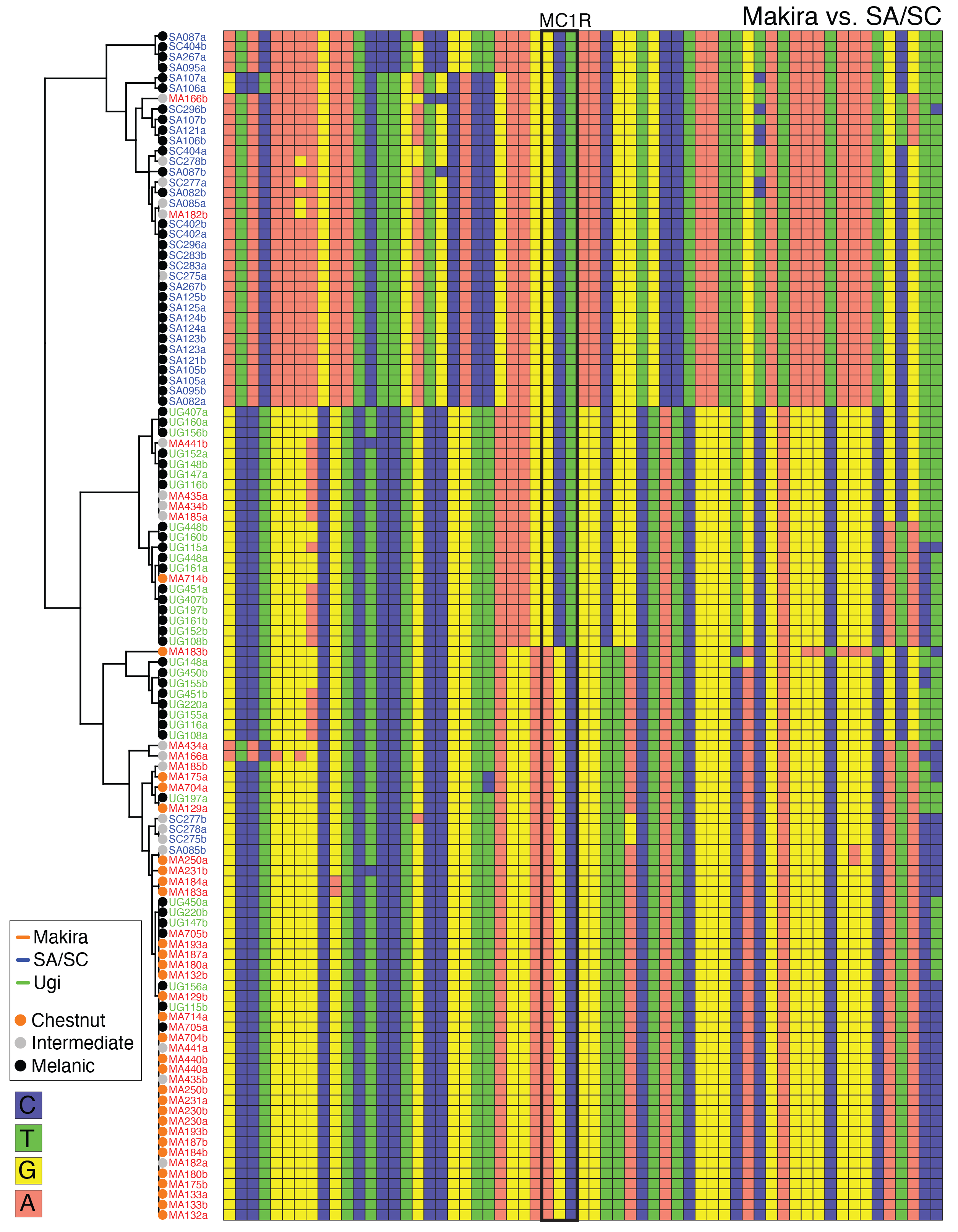

Supplement: S4 Fig — Clustering of haplotypes obtained from the association peak on contig 400. Phased genotypes for the 61 SNPs located in the association peak on contig 400. Rows represent single chromosomes, therefore individuals are represented twice in the clustering tree on the left. The four nucleotides, the collection locality and the coloration phenotype are color-coded as indicated at left. The three SNPs within the MC1R coding region are indicated with a black rectangle. All individuals from SA/SC contained at least one haplotype in the region delimited by the SNPs with significant association scores around MC1R that differed from the one present in most individuals from Makira and Ugi. All the melanic individuals from SA/SC possessed two copies of this haplotype, while the four individuals with intermediate coloration possessed one of each, as was the case for two of the six individuals with intermediate coloration from Makira. All melanic individuals from SA/SC carried two copies of the derived Asn119 mutation, while all but one of the chestnut-bellied individuals from Makira had two copies of Asp119. The individuals with intermediate coloration (from either Makira or SA/SC) were heterozygotes for this coding mutation. The exception to this pattern was a single chestnut-bellied individual from Makira (MA714), which was a heterozygote yet was scored in the field under heavy molt and may have been incorrectly classified as having a chestnut belly. Finally, the derived Asn199 mutation existed primarily on the haplotype background found on SA/SC, but also to a lesser extent on the haplotype background found on Makira and Ugi. We note that the chestnut-bellied MA714 bird carried the Asn119 mutation on the most common haplotype background observed on Makira. The birds from Ugi, which are all melanic, were sometimes homozygous for either allele or heterozygotes. (TIF) [file pgen.1010474.s006.tif]

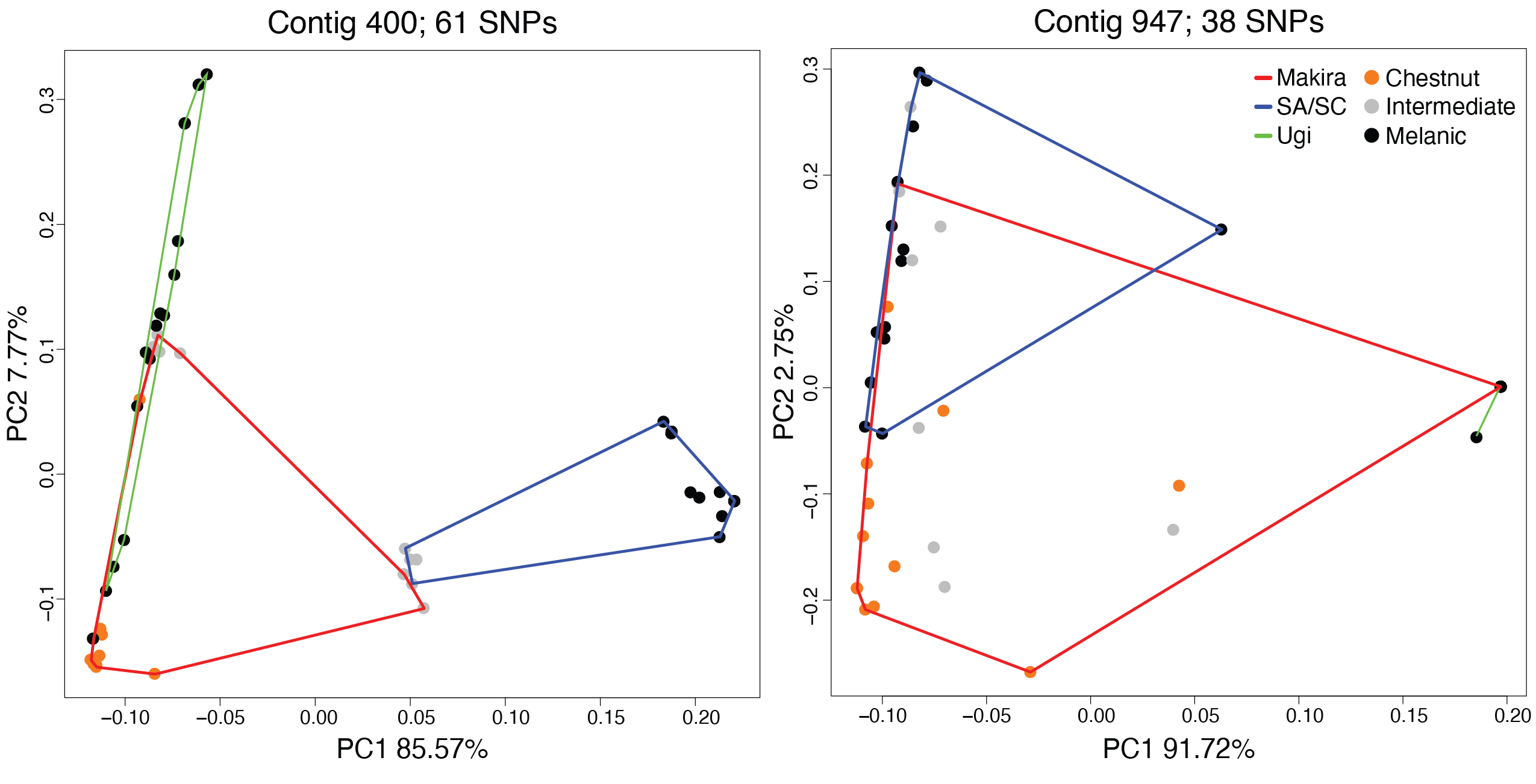

Supplement: S5 Fig — Principal component analyses derived from the SNPs within the association peaks. PCAs from the variants within the association peaks on contig 400 and contig 947. Coloration phenotype and the island where individuals were sampled are color-coded. (TIF) [file pgen.1010474.s007.tif]

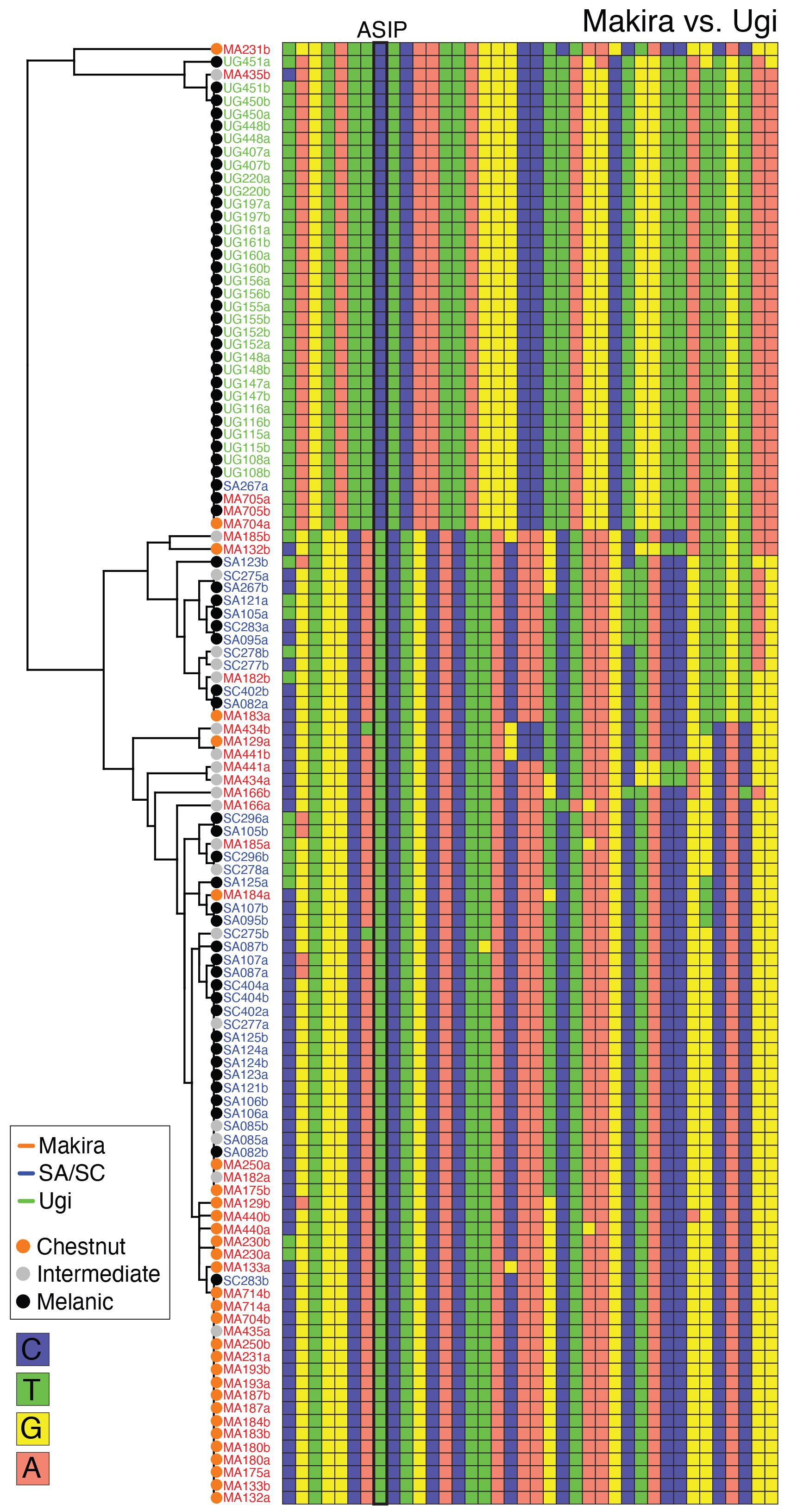

Supplement: S6 Fig — Clustering of haplotypes obtained from the association peak on contig 947. Details as in S4 Fig. Ugi individuals had two copies of a haplotype that was different from the one present in SA/SC and Makira individuals. The only melanic individual from Makira (MA705) also carried two copies of the derived Ugi haplotype. We found a few heterozygote individuals but there was no obvious pattern with respect to their coloration. (TIF) [file pgen.1010474.s008.tif]

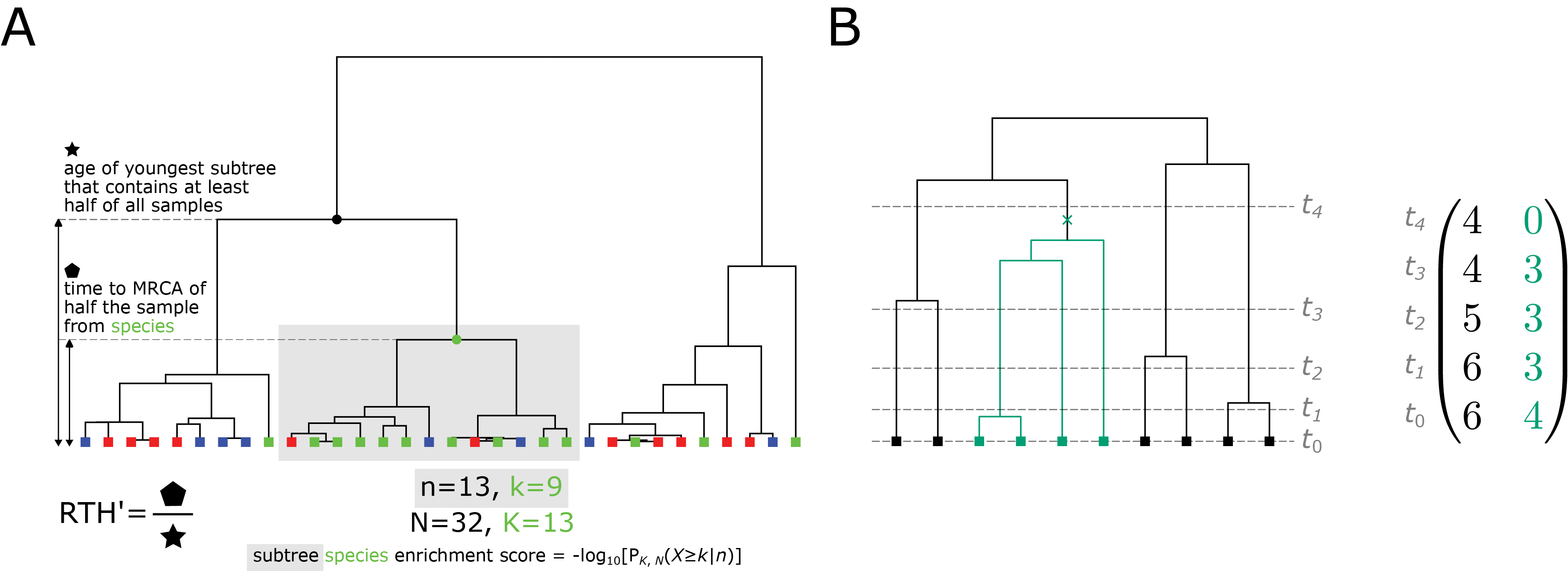

Supplement: S7 Fig — Statistics and feature encoding of the genealogy. A. RTH’ tests for the reduction in within-species TMRCA and is defined as the ratio between the TMRCA of half of the samples from a given species and the age of the youngest subtree that contains at least half of all samples. The species enrichment score tests for species differentiation in local trees and is defined as the maximum score associated with a given species in a subtree of the full genealogy. For a subtree, the species enrichment score is the probability of observing the number of samples of a particular species in that subtree under a hypergeometric distribution. Here the coloring of the leaves indicates hypothetical species and the example illustrates the statistics with respect to the green species. B. Genealogical features for the SIA model consist of the number of lineages in the genealogy at a set of discrete time points (t0, t1, …). The time points are chosen in an approximately log-uniform manner resulting in finer discretization of more recent time scales. In addition, when encoding the genealogical features at a particular site of interest, we encode separately the counts of ancestral (shown in black in the example) and derived (shown in aquamarine) lineages. The aquamarine cross indicates the branch where the mutation occurred. (TIF) [file pgen.1010474.s009.tif]

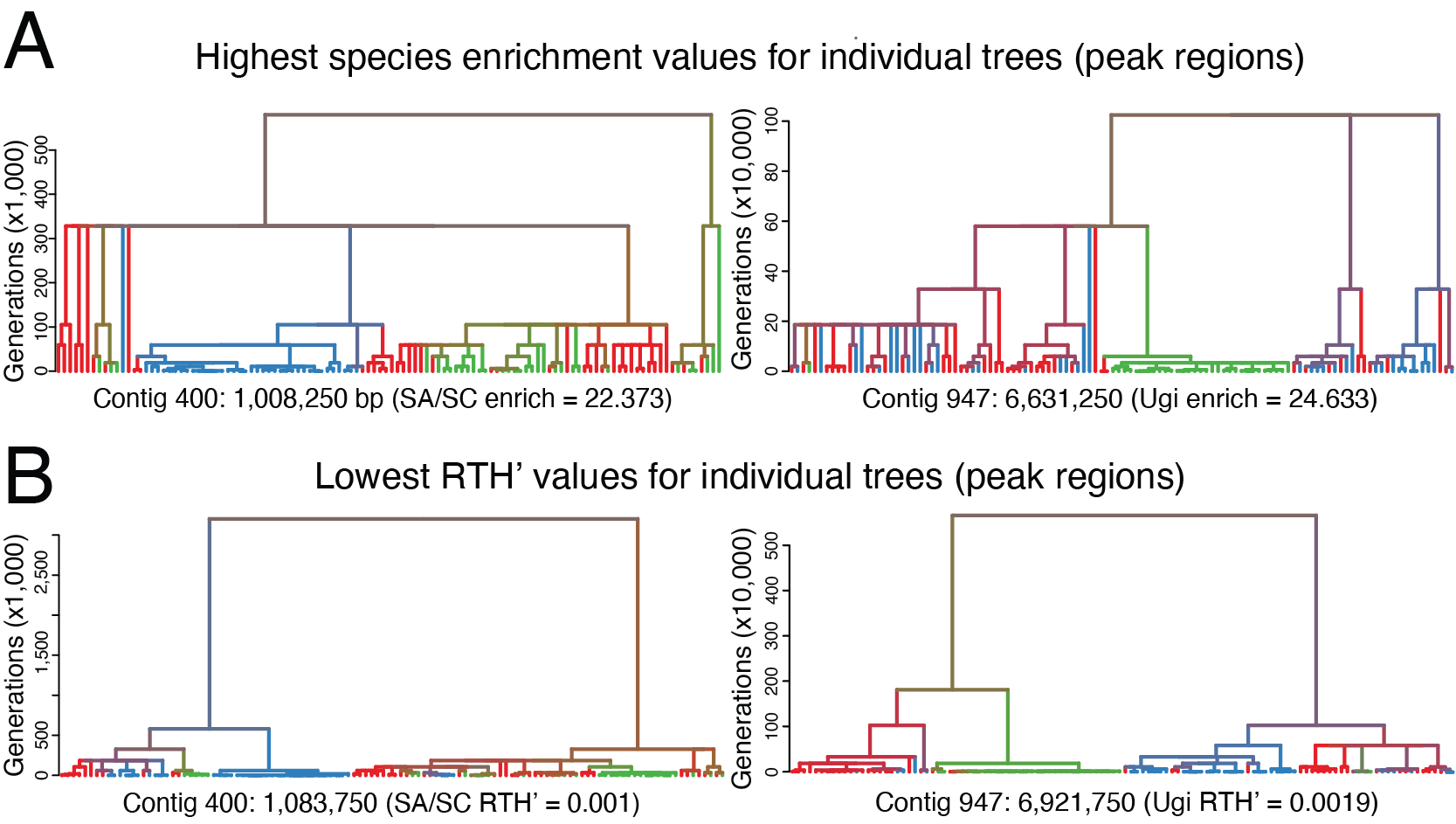

Supplement: S8 Fig — Highest species enrichment scores and lowest RTH’ values for peak regions on contigs 400 and 947. Representative trees from the peak regions which show extreme values of species-enrichment (A) and RTH’ (B) for SA/SC and Ugi. (TIF) [file pgen.1010474.s010.tif]

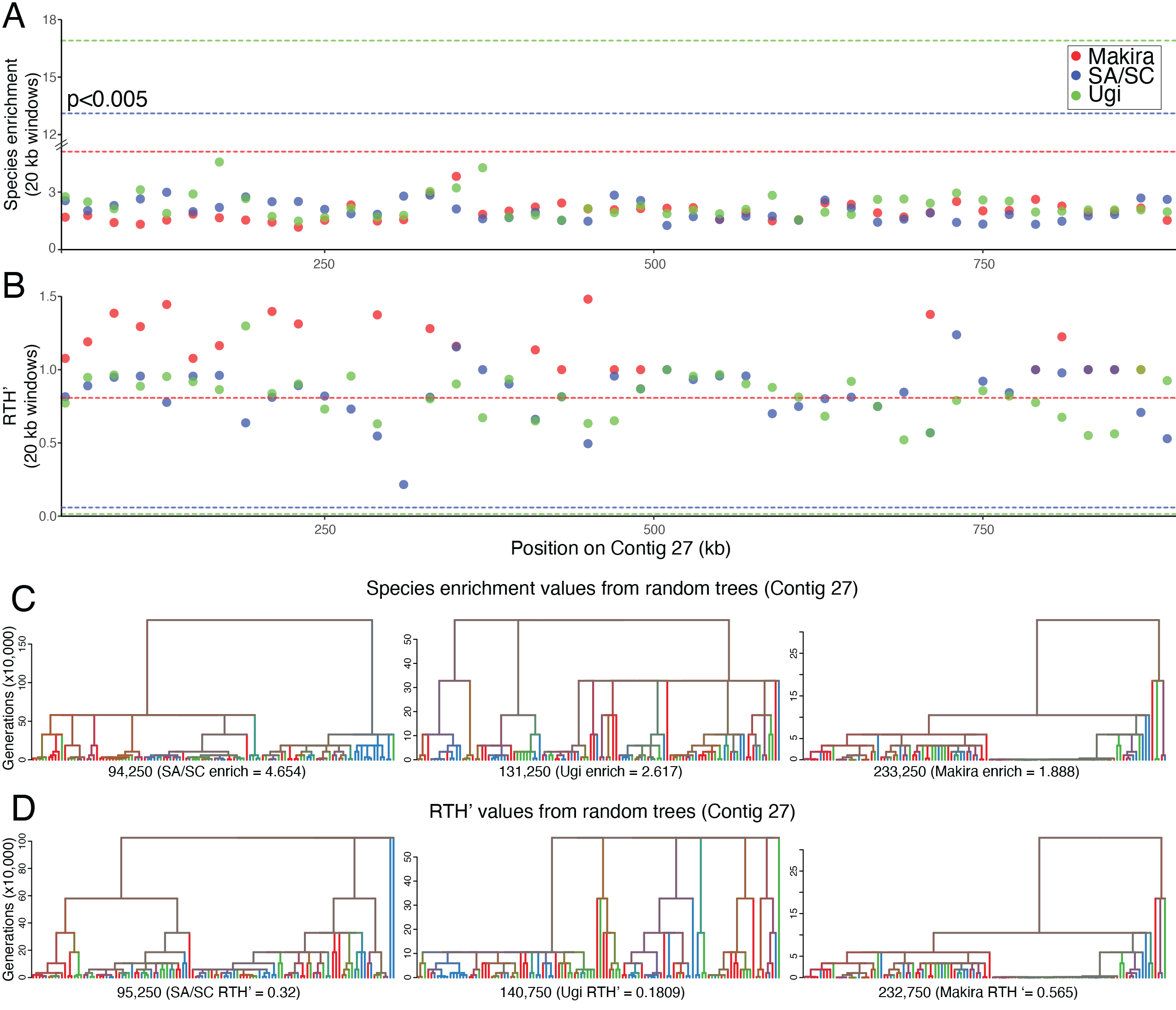

Supplement: S9 Fig — Species enrichment scores and RTH’ values for a control contig. Plots showing species enrichment (A) and RTH’ (B) in 20 kb windows along contig 27. Horizontal lines show species-specific levels of statistical significance (p<0.005). Trees obtained from random positions on contig 27 (each position is shown under the tree) indicating species enrichment scores (C) and RTH’ (D) values for each population. (TIF) [file pgen.1010474.s011.tif]

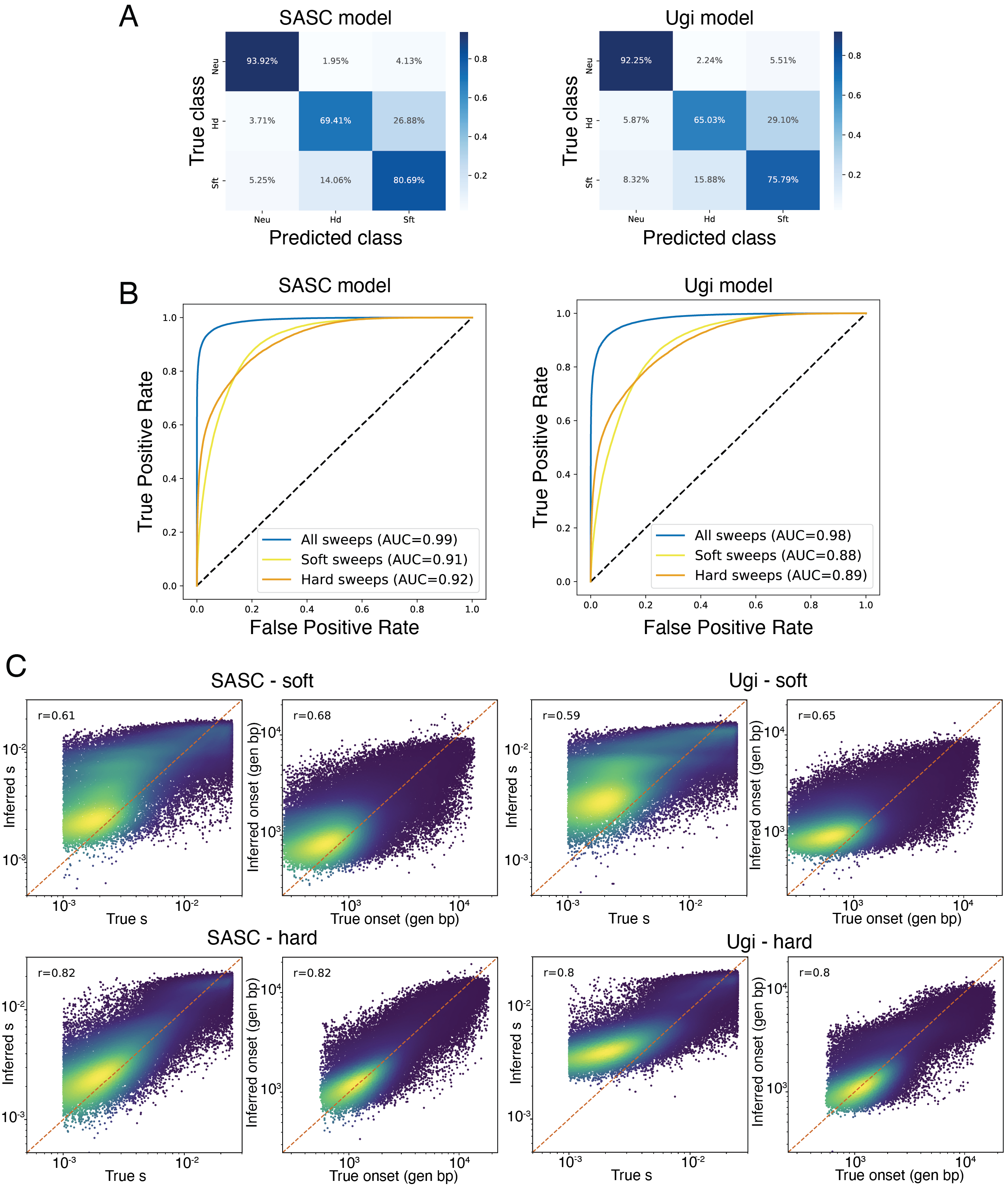

Supplement: S10 Fig — Benchmarking of the SIA models. A. Confusion matrices generated by applying the SA/SC and Ugi models to simulated data. Instead of applying a specific probability threshold, the predicted class was identified as the one with the highest probability according to the model. Under this maximum likelihood classification scheme, both models perform best distinguishing neutral from selected sites, and moderately when identifying soft or hard sweeps. B. One-versus-rest (OvR) receiver operating characteristic (ROC) curves of the model classification performance on simulated data. The models perform very well on distinguishing sweeps, and less so on precisely identifying hard or soft sweeps. C. Comparisons between true and inferred selection coefficients and time of selection onset (expressed in generations before present) for SA/SC and Ugi models trained to detect soft or hard sweeps. Models trained to predict hard sweeps generally perform better than those trained to predict soft sweeps, which tend to overestimate small selection coefficients and recent selection onset times. (TIF) [file pgen.1010474.s012.tif]

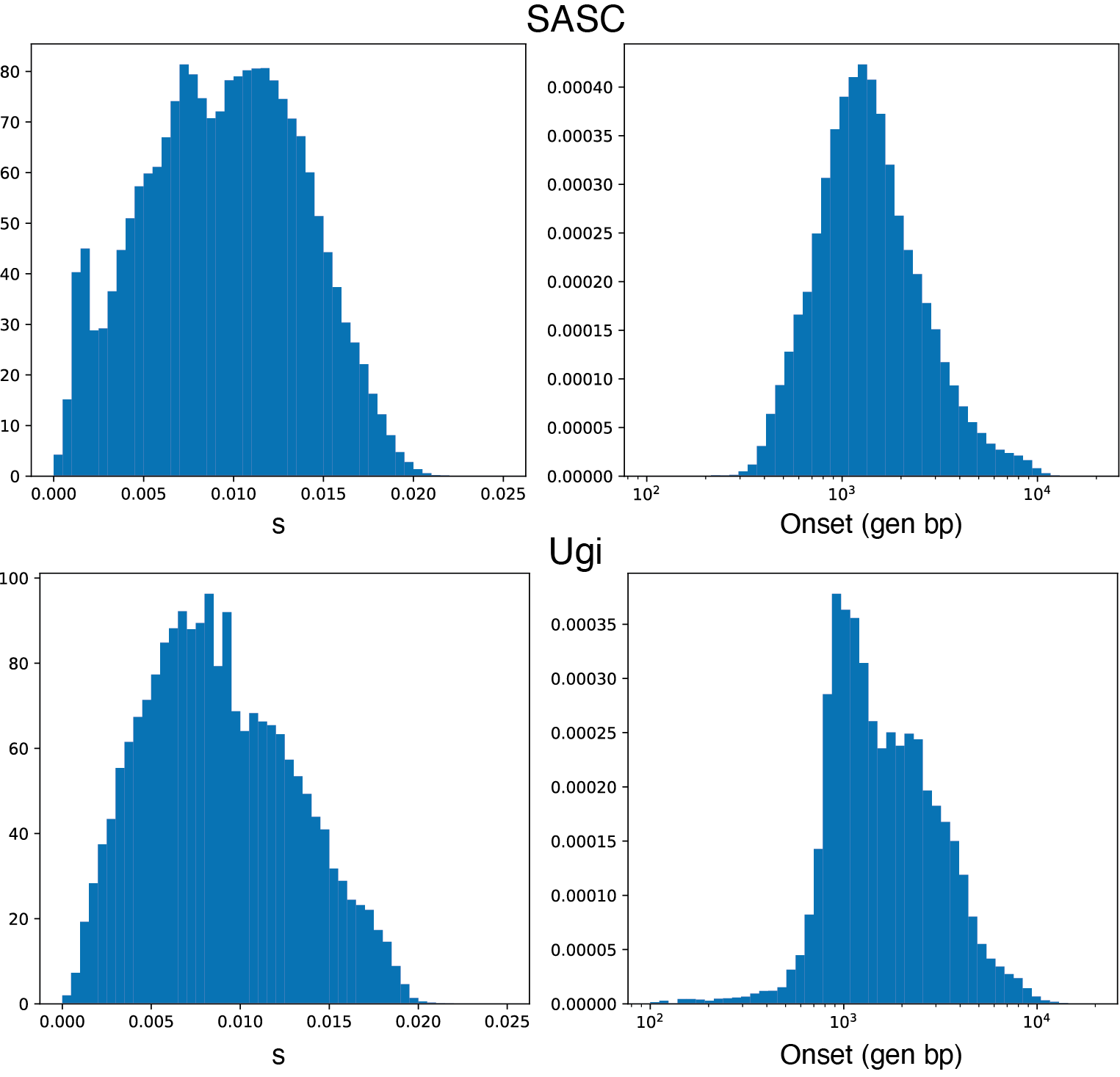

Supplement: S11 Fig — Genome-wide estimates of selection coefficients and time of selection onset. Predictions are based on all the variants from the 190 scaffolds longer than 100kb. (TIF) [file pgen.1010474.s013.tif]
