# Supplementary material for: Selective sweeps on different pigmentation genes mediate convergent evolution of island melanism in two incipient bird species
Source: PLoS Genet. 2022 Nov 1;18(11):e1010474. doi: 10.1371/journal.pgen.1010474 (PMC9624418; doi:10.1371/journal.pgen.1010474)
Supplement: Attachment — Submitted filename: Review_PloS_Genetics_2022.docx [file pgen.1010474.s015.docx]

**Summary**

In this article, the authors revisit a classic example of melanic colour variation in the chestnut-bellied flycatcher. They confirm that selection on two major pigmentation genes, *ASIP* and *MC1R*, is responsible for the independent emergence of melanic forms on Ugi and Santa Ana/Santa Catarina, respectively. They estimate the onset of selection and its strength, showing that melanism is likely older on Ugi than SA/SC, and under strong (s~0.01) selection. Their analyses take the demographic history into account. This is an excellent analysis using state of the art methods. The manuscript is clear and straightforward, and clearly shows the interest of this system for evolutionary biologists. There are many good ideas in terms of analyses, and the authors apply several descriptive (H1/H2, haplotype structure) and more complex (ARGs, machine learning) methods to interpret their data. Some results tend to be left aside (e.g. peaks of association on the Z chromosome, variant annotation near *ASIP*), as the authors mostly focus on candidate genes/variants that are already known in the system. This is not necessarily a problem in my opinion, but it should be made more explicit, and a brief plan for further analyses could be laid down at the end of the Discussion.

**Major comments**

Association analysis:

An intriguing finding is the peaks on the Z chromosome (mentioned L148, it would be good to highlight these peaks on Figure 2). Did the authors consider the sex of individuals when running their GWAS on Z scaffolds, since it could bias association tests? Assuming no bias due to heterogametic chromosomes, could this pattern reflect accelerated divergence on the Z due to lower effective population sizes, loci involved in reproductive isolation, or other loci involved in melanism? Are these variants in LD with variants near *ASIP*?

Given the clear genetic structure, it may be interesting to use the demographic models to estimate the expected distribution of p-values for association for autosomes and sex-linked markers. The *p*-values seem quite low given the sample sizes, even for discrete traits with a simple genetic determinism. The authors mention Q-Q plots but I could not find them in the manuscript. Did the authors correct for genomic inflation (*λ*)?

Another intriguing aspect is the discrepancy between association and selection for *ASIP*. It could be indeed due to fixation/soft sweeps (as briefly mentioned L247-248). Authors run two GWAS analyses, grouping individuals from the main island with individuals from Ugi, then SA/SC. Given the strong genetic structure and demographic history, I am unsure that running two GWAS is the best option. A complementary method here could be BAYPASS (Gautier, 2015), to systematically compare allele frequencies across all populations/phenotypes at once in the analysis while correcting for relatedness between populations. You could then compare the association signal between the two analyses and their consistency. This could possibly eliminate some of the noise, or reveal signals that are less clear with the GWAS.

Onset of selection:

I would recommend that the authors show the TMRCA for each population/phenotype (although this information is partially conveyed in the trees shown on Figure 3). This may help interpreting the lack of selection signal for associated variants near *ASIP*, and support their interpretation about an older, completed sweep. It may be interesting to examine the TMRCA of haplotypes found associated with melanism, to make the most of the information contained in the ARG.

In the case of *ASIP*, it seems likely that several cis-regulatory variants may play a role. If the authors intend to further investigate the system (and others) in a future work, I suggest Relate (Speidel, Forest, Shi, & Myers, 2019) to estimate genealogies along the genome, in combination with CLUES (<https://github.com/standard-aaron/clues>, (Stern, Speidel, Zaitlen, & Nielsen, 2021)) to test for polygenic selection.

Functional variants (*ASIP*):
L159-160: It would be worth mentioning here the more distant peaks already (close to *EIF6* for example), as they may well correspond to important cis-regulatory variants. Studies in humans (among others) have shown signals of association quite far from *Agouti*. Moreover, given that differences in pigmentation are mostly found on the belly, it may be interesting to check position of GWAS hits relative to ventral promotor that have been described in, e.g., quail (Nadeau et al., 2008), if homolog sequences can be found.

It might be worth to check for rearrangements (e.g. with DELLY? https://github.com/dellytools/delly) in the region. The presence of many associated SNPs at the end of the contig (i.e. near a region difficult to assemble) could be consistent with some sort of inversion. I understand the limitations of short reads for calling large structural variants, and leave it to the authors to decide whether this is feasible.

**Minor comments**

L193: I would be a bit more cautious when discussing the efficiency of SIA. It is fine given that you have clear candidates for selection, but this would not be very satisfying for a naïve genome scan for selection. Confusion matrices suggest that the classifier cannot distinguish soft and hard sweeps that well, and the false positive rate for sweeps is around 8%. On Figure S9, what is the probability threshold to classify a window as neutral, hard sweep, soft sweep? How does the matrix change when changing the threshold for, say, p(neutral)?

L208: Maybe show differentiation (e.g. F_ST_) between islands for SNPs that SIA identifies as under selection. Maybe show haplotype extension, for this region compared to *MC1R*. If it is indeed an ancient completed sweep, haplotypes should be shorter. The package rehh may be able to run quickly and works with unphased data: <https://cran.r-project.org/web/packages/rehh/vignettes/rehh.html>

L336: Explain why you mask the genome before gene annotation (to avoid annotating TEs as genes I assume). This can be useful for readers not familiar with genome assembly.

L438: Mutation rate seems a bit low (10^-9^) compared to other rates that are closer to 2.10^-9^/year. Briefly mention how this can change your demographic estimates.

I hope you find these comments constructive. Congratulations for this nice work.

Yann Bourgeois

**References**

Gautier, M. (2015). Genome-Wide Scan for Adaptive Divergence and Association with Population-Specific Covariates. *Genetics*, *201*(September), 1555–1579. doi: doi:0.1534/genetics.115.181453

Nadeau, N. J., Minvielle, F., Ito, S., Inoue-Murayama, M., Gourichon, D., Follett, S. a, … Mundy, N. I. (2008). Characterization of Japanese quail yellow as a genomic deletion upstream of the avian homolog of the mammalian ASIP (agouti) gene. *Genetics*, *178*(2), 777–786. doi: 10.1534/genetics.107.077073

Speidel, L., Forest, M., Shi, S., & Myers, S. R. (2019). A method for genome-wide genealogy estimation for thousands of samples. *Nature Genetics*, *51*(9), 1321–1329. doi: 10.1038/s41588-019-0484-x

Stern, A. J., Speidel, L., Zaitlen, N. A., & Nielsen, R. (2021). Disentangling selection on genetically correlated polygenic traits via whole-genome genealogies. *American Journal of Human Genetics*, *108*(2), 219–239. doi: 10.1016/j.ajhg.2020.12.005
