# Supplementary material for: Selective sweeps on different pigmentation genes mediate convergent evolution of island melanism in two incipient bird species
Source: PLoS Genet. 2022 Nov 1;18(11):e1010474. doi: 10.1371/journal.pgen.1010474 (PMC9624418; doi:10.1371/journal.pgen.1010474)
Supplement: Attachment — Submitted filename: Response to reviewers.docx [file pgen.1010474.s016.docx]

September 14, 2022

Dr. Nicolas Bierne

Academic Editor

PLOS Genetics

Dear Dr. Bierne,

Thank you for your letter from August 11^th^ regarding our manuscript PGENETICS-D-22-00667 entitled " Independent selective sweeps on pigmentation genes mediate parallel evolution of island melanism in two incipient bird species”. We found the comments and suggestions on our manuscript very helpful and have uploaded a revised version. This version includes analyses that are presented in two new and one modified supplementary figures, an additional figure in the text (moved from the supplement), and a new supplementary table. We have also expanded some sections of the text (e.g. the discussion) following the suggestions that we received. We provide detailed point by point responses to the comments below and include a copy of the manuscript and the supplement with the changes tracked (note that line numbers in the response to reviewers refer to the tracked version). Finally, we also provide a spreadsheet with the numerical data underlying the main figures and a few potential cover images in case the manuscript is accepted for publication.

Sincerely,

Leonardo Campagna on behalf of all co-authors.

Thank you for your submission to PloS Genetics. We have received three thoughtful reviews of your manuscript entitled “Independent selective sweeps on pigmentation genes mediate parallel evolution of island melanism in two incipient bird species”. The referees are globally positive and expressed mainly minor concerns, though important to account for. I’d ask you to account for all these concerns in a revised version. Two referees were surprised you did not investigate further, nor discuss, the other peaks showing up in the GWAS, and especially the one of the Z chromosome. Referee 2 asks for more details about SNP filtering. Referee 3 suggests additional analyses that you can do, or alternatively you can argue why you did not. They all three provide additional comments that should help you to clarify some points of the paper. The paper has been written to be short but now it has been transferred to PLOS Genetics you have more space to explain complex analyses in more detail.

We thank the editor and reviewers for their time and effort to improve our manuscript. Please see our detailed responses below.

On my side, I also was very interested by your work and I learned a lot with the reading of your ms and the literature I had to catch up to understand it. It is well written and the results are important findings. The method is appealing. I would ask you the followings:

- Please clarify the novelty of your finding. Given it was already strongly suspected that two different genes were involved in melanic evolution in the different satellite islands, independence is rather a confirmation than a new result. In addition, the evidence for a selective sweep at ASIP on Ugi is rather weak (and/or not clearly discussed). The novelty is on the evidence of selection, but mainly at MCR1 on SA/SC, and also in the method. I think the title is somewhat misleading (the “s” in “sweeps”) and could be reworked to better fit the originality of your work and still attracts readers.

We have expanded the discussion on the evidence of a sweep at *ASIP* in the Ugi population which before was limited to the SIA result but now better integrates the support that we found through other analyses (e.g., new Fig. 3). We therefore believe that there is evidence for independent sweeps on *MC1R* and *ASIP* and that these lead to convergent melanism (we now avoid using the term parallel as explained below). We believe this title focuses on the main finding that independent selective sweeps are responsible for the convergent evolution of this phenotype. We also state the main novelty of our findings in the first paragraph of the discussion.

- Please clarify what you mean with “independent” and “parallel” and use these terms with care. Given the molecular mechanism is different in Ugi and SA/SC, independence is obvious and only phenotypic evolution is parallel not molecular evolution. For instance the title on line 165 “MC1R and ASIP show signatures of parallel selective sweeps”, is an unusual use of the term “parallel” (although possible if well defined/explained, given “parallel” is often a catch-all term).

We agree with the editor that the use the term “independent” is obvious in the contexts that we use the term (e.g., “independent mutations”, “independently derived”). However, the term parallel generated confusion as in the literature it tends to be used to refer to the phenotype, the molecular targets or both. We agree that in the case of our study system it only applies to the phenotype and therefore we have replaced parallel for convergent or deleted it in contexts where it was not needed.

- The novelty is in the method and the data type used (ARG-derived) while you devoted few space to explain them, relying on previous publications. I think a cartoon (e.g. as a sup file) is needed to explain “species enrichment” and “RTH’”, in addition to the example of figure S6. Species enrichment could be called differently as you use it for population genetics, and it could be described with other words (eg “population” instead of “species”). I didn’t immediately understand what you meant with species here. This ARG-derived statistics is calculated for a sample/population, but depend on the all ARG on the whole sampling as far as I understood. It seems similar to a population-specific Fst, and somehow informs about genetic differentiation (hence the peak at ASIP in fig 3). “RTH’” (please recall RTH means “relative TMRCA half-life”) is well suited to identify diversity trough but I find it difficult to have intuition about it’s behavior as, again, the coalescence of all haploid samples is included in the calculation. SIA is a new method you developed and has not been often used. It would need more explanation. Especially, what exactly are these features from the local genealogies extracted from the ARG? Given it’s not super clear in the SIA paper, I would find it useful for the reader if you could better describe them (again a cartoon as sup file?). In addition, you said “SIA uses features of single-population genealogies of the satellite island population” (line 553) and I’d like you to better explain and elaborate on the possible implications (e.g. in comparison with the study of “species enrichment” and “RTH’” in the previous analysis).

Thanks for the suggestion! We added a supplementary figure (Fig. S7) to illustrate how the species enrichment score and RTH’ are calculated as well as how the genealogical features used by SIA are created. We also updated the opening paragraph of the SIA method section (lines 701-710) to better explain the motivation of using SIA in addition to the species enrichment score and RTH statistics. Finally, we have clarified the meaning of each of the ARG-derived statistics.

- I was pleasantly surprised to see you used two-pop simulations in SIA. The reason is that local hard sweep in a subdivided population could easily be classified as soft sweep if a classifier is trained with single pop simulations (after fixation unswept haplotypes introgressed from the continent population into the shoulders of the swept region of the island population). I think you might insist more on this point.

We now explicitly mention the advantage of the 2-pop simulation setup in the methods (lines 733-739).

- I found the discussion of the ASIP sweep (if a sweep really happened) on Ugi a little confusing. SIA prefers a neutral model at ASIP, so you discuss the little evidence of soft sweeps 300-400 Kb away in the flanking regions. What’s the idea? Regulatory regions as proposed by referee 3? what’s the differentiation? not as strong as at ASIP according to species enrichment (indeed a good old Fst Manhattan plot could be useful here, as sup file). Or is this the shoulder of the ASIP sweep (hence a soft sweep signal)? How does this region recombine? Could a chromosomal rearrangement be suspected (aslo suggested by referee 3)? If you discuss the age of the soft sweep it means you have selection on these flanking regions, not at ASIP. Please clarify each hypothesis, explaining when you consider selection is targeting Ile55Thr and when you consider selection is targeting other variants away on the contig. In addition, is an old fixation at ASIP-Ile55Thr and footprint lost compatible with low RTH’ at ASIP? Looking at the zoom around Ile55Thr in figure S5 one would rather bet on a recent hard sweep.

We have expanded the discussion on the evidence of selection and the sweep on *ASIP* in the Ugi population. There are many lines of evidence suggesting there has been selection on this region. First, we generally observe elevated differentiation within the association peak compared to outside of the peak (Fig S3B). We also see elevated species enrichment, low RTH’, low nucleotide diversity and a combination of high H12 and intermediate H2/H1. We have moved the Tajima’s D, nucleotide diversity and H-statistics figure to the main manuscript (Fig. 3). What we think is happening is that the GWAS hits for the *ASIP* contig (including the position leading to Ille55Thr) are in high LD (hence the pattern in Figure S6) and fixed in Ugi. The simulation used to train SIA allowed variants to fix, yet it becomes harder to detect signatures of selection when the sweep is old (and SIA is applied to the Ugi data alone). As we mention in the discussion the mutations in and around *ASIP* were estimated to occur 174 K generations before present, and SIA was not trained to detect selection older than 20 K generations, and rarely identified selection older than 10 K generations. We believe that for these reasons we were unable to detect selection in this region using SIA despite the other evidence mentioned above. SIA did detect selection on specific positions that were not GWAS hits but were in the regions that produced the outlier statistics mentioned above. It is possible that these are more recent mutations (indeed SIA estimated them to be in the order of 12-14K years old) that could involve cis-regulatory regions.

- Figure S7: “Circles represent statistics derived from 100-SNP windows (left panels) or 5 kb windows (right panels)” Are left panels lacking?

We have replaced the sentence with: “Dots represent statistics derived from 5 kb windows (Tajima’s D vs. nucleotide diversity) or 100-SNP windows (H12 vs. H2/H1), and are color coded based on the population of origin.” The window size is different for each type of analysis. Note that this figure was now moved to the main manuscript (Fig. 3) and panels are labelled with letters.

- Discussion: Melanic mutants segregate with low or negative s during ~100K generations and then s switch to 2% 500 generations ago. Why melanic evolution takes place now? What happens with these satellite islands, are they new? Or why would sexual selection switch on suddenly? Does melanic evolution take place recurrently in many systems and you arrived at the good pace now in this system? I'm lacking of context here.

Our analysis indeed seems to suggest that the coding mutation on *MC1R* existed at low frequencies for some time until the selection regime changed, and then it rapidly swept on SASC. For instance, it is possible that melanism is favored by female choice (sexual selection), in which case the preference for melanic plumage may need to reach a frequency threshold before positive selection can take off. Therefore, female preference could evolve and eventually drive the evolution of melanism. We have added this to the discussion and provide a reference for context (Kokko et al. 2002). The satellite islands are comparatively old (millions of years) yet it’s likely that cyclones can drive the small populations to local extinction periodically.

Overall, these are matter of clarification and discussion and I'm convinced you will address my and referees concerns easily in a new version. I’m looking forward to reading it.

Best regards,
Nicolas Bierne

Reviewer #1: Leonardo Campagna and colleagues build a new reference genome and collect resequencing data of different Chestnut-bellied Flycatcher populations in the Solomon Islands to study the origin and evolution of melanic phenotypes. They mostly build from a previous study (Uy et al. 2016. Proc B) that already demonstrated the unique evolution of melanism in the two separate island populations. However, in this study, they identify the selective sweeps around the causative loci and from these sweeps estimate strength and timing of selection.

I really enjoyed reading this manuscript. It is clearly written and it presents interesting results. For example, the authors use nifty statistics to identify patterns of selection and use machine learning (trained with their demographic inference models) to classify soft and hard sweeps as well as the timing and strength of selection. To me, that is novel and this study will provide guidance for other studies to do this. As the authors note themselves, it was also surprising to me that at the geographic scale, the potential dispersal distances of the birds, and with the mutations being present at low frequency in Makira that the satellite populations adapted using different mutations. I think this study is thus a compelling example that shows that evolution from standing genetic variation does not have to be the default.

We thank the reviewer for the encouraging comments.

My main comment is that I am still curious about the other peaks showing up in the GWAS, including the Z chromosome. Is the lack of any color pattern genes in the additional GWAS peaks enough argument to dismiss them? What if these included genes that have not been linked to color or well characterized? If so, it might be important to discuss the additional peaks further.

We have now added a supplementary table listing the genes in each association peak and the contig/chromosome to which they belong (new Supplementary Figure S1). We don’t know of a link between these genes and melanism/coloration but the reviewer is correct that they may have one that is yet unknown. Alternatively, they may be involved in other aspects of the differences between the Ugi and Makira populations which may covary with the differences in coloration. We now mention these possibilities and provide the table in case in the future a connection can be made between some of these genes and phenotypes, and our data becomes useful for further analyses (e.g., meta-analyses).

We also mention that the Z sex chromosome is known to evolve faster in birds and provide a reference (for various reasons including a wider range of conditions that allow a mutation to increase in frequency, a slightly higher mutation rate, and increased genetic drift), which is consistent with our finding of three out of seven peaks being on this chromosome. However, as with the other GWAS hits, we can’t make a direct connection with phenotypic differences.

L120 and L148 describe outlier results on the Z chromosome. This chromosome may have a proportionally important role in speciation, but no further mention is made or explanation is given.

We now mention that this is consistent with the fast-Z effect in birds and provide a citation. It is hard to know if these differences are due to drift or selection which is unrelated to coloration.

Additional comments:

The results are mostly clear, but some parts, I think, would benefit from adding some details on the used methodology (see below for specific sections), rather than having to look for them in the methods.

We have added brief descriptions of the methods while trying not to alter the flow of the paper.

L54-55: These are examples of insular systems. This sentence might benefit from adding a motivation of focusing on insular systems.

We selected these examples primarily because they included recent examples of convergent evolution in vertebrate species, which are the most relevant to our study. It is true that they are examples of insular systems, yet this is somewhat of a consequence of our ability to study convergent evolution in vagile species on islands. We therefore chose not to take away the focus from the main point which is that recent examples of convergent evolution allow us to study the evolutionary forces and molecular mechanisms behind this process.

L66: Please clarify the data used in the phylogenetic analysis.

Done (Line 76).

L125: Please provide some details on the demographic reconstruction here. What tool and what type of data was used?

Done (Lines 136-137).

L130: How was gene flow inferred? Please elaborate on what type of signals are looked for in the data to provide confidence in these estimates.

We clarified that this was done in G-PhoCS and briefly describe the methods in the relevant methods section (Line 573). Reference 63 (section S4.2 of the supplement) explains the method in greater detail.

L183-184: “resulting from” instead of “derived from”? Or “statistical outliers” instead of “outlier statistics”?

Fixed (Line 240).

L193: How was this data simulated, in short? (I would at least mention the program and that parameters were derived from the demographic inference model).

Done (Lines 249-251).

L198-200: Can confidence intervals be provided for the selection strength and time estimates?

We applied dropout at inference time to produce confidence intervals for the selection parameter estimates (see updated Fig. 5 and lines 765-769). The theory behind this technique is explained in reference 74.

L421: Please provide some more information on how G-PhoCS works.

ok.

L469: Do the two haplotypes explain the soft sweep?

This sentence refers to the result from phasing the haplotypes in the candidate regions and simply indicates that the phased data results in two haplotypes per individual.

L537: What is the motivation of down sampling the effective population size? I assume computational feasibility? Could this not strongly impact the training data and inferred results on selection strength and timing?

We have clarified this point in the manuscript (see Lines 731-733 and 739-741). Scaling down the population sizes requires scaling other parameters (e.g., selection or divergence time) appropriately, and so does not impact the inferred results.

Reviewer #2: In this manuscript the authors identify and characterize independent soft selective sweeps associated with a melanism in Monarcha castaneiventris ugiensis. They show that the origin and timing of the onset of selection for a blabk belly differ between the subpopulations in the islands of Ugi and Santa Catalina / Santa Anna. This is an original study and it is noteworthy that this is a case of convergent selection although the two melanic populations share a common origin. The conclusions of the study rely on strong genetic evidence and I have a single minor comment about the methodology (my point 1)

We thank the reviewer for helping improve our manuscript.

1- SNP filtering may affect the results and the statistics: the authors sometime discarded SNPs with an absolute MAF (minor allele frequency) lower than 8. In some cases this filtering was not applied but this choice is not clearly stated. This filtering affects nucleotide diversity (pi) and the expected distribution of Tajima's D (which will be skewed towards positive values) this may partly explain the results shown on fig. S7.

We have now clarified that we use the dataset with the MAF filter unless otherwise stated. The exceptions are for the demographic modeling in GPhoCS and for the selection inference in SIA, where we did not want to bias our analysis by only using alleles that segregate at higher frequencies. No we also use this dataset for calculating Tajima’s D, nucleotide diversity and H-statistics.

The reviewer is correct to point out the effect of this filter on pi, Tajima’s D and the H statistics and in response we have re-done these analyses without the filter. Indeed, this filter had a general impact on the statistics and by including low frequency alleles the H statistics show evidence of relatively soft sweeps (high H12 but intermediate H2/H1 values) for both contigs (400 for SASC and 947 for Ugi). We now also observe slightly higher values of nucleotide diversity, and the distribution of Tajima’s D values is no longer skewed towards positive values. We have revised this figure (now in the main text as Fig. 3) and our interpretation of the patterns. However, because of the windowed nature of these analyses, we don’t wish to place too much focus on these results and are generally cautious about their interpretation.

2- Sampling: The sample described lines 283-292 is not clear. Please double check that the text conforms to the suppl. table s1.

Apologies for this mistake. We fixed these inconsistencies (section 1 of materials and methods).

3- Genetic differentiation of M. c. megarhyncus and M. c. ugiensis: Most of the results stand in the PCA analysis (fig 1D) where the first axis clearly separates the two M. c. ugiensis populations. It is also clear that Ugi is more distant than SA/SC from Makira, which is confirmed by the FST values (Fig 1F). Note that FST are computed between populations not between individuals (lines 381-382 in the main text).

Agreed, we replaced “individuals” with “populations” (Line 519).

4- Figures: Notice that the figures are often tiny and hard to read.

The figures are made to be legible on letter size paper and the fonts are at least 5-6 point, yet we can provide specific revisions once they are formatted to their final size if the manuscript is accepted for publication. For the initial submission they were embedded into the word file and this may have made them harder to read.

-- The color choice (Ugi green / Makira red all along the manuscript) may be especially challenging for colorblind readers.

It seems like the difference in saturation and contrast in each color and not just the hue can help them be distinguish under different conditions of color blindness.

We checked our main palette using the website indicated below and it seems like the combination of colors we used can be distinguished under the most common conditions (follow the link to see how they may be perceived).

<https://davidmathlogic.com/colorblind/#%236ABD45-%23ED2224-%233A53A4>

-- On figure 1G, confidence intervals are hardly visible on the gray background and invisible on black.

We’ve increased the width and contrast of the lines indicating the confidence intervals in this panel.

-- The curves in suppl fig S2C-S2F are assignment probabilities of SA/SC ancestry or Ugi ancestry, because these curves were smoothened with gglot2,unfortunately leading to probabilities that may appear negative or >1 on some intervals, which is inconsistent with a probability.

The reviewer is correct to point out the effect of the smoothing curves, and particularly the confidence intervals around these curves, and we clarify in the legend that this is the reason why the plots extend beyond 0 or 1. We tried clipping the plots, shading the regions beyond the [0,1] interval or indicating the interval with horizontal lines, however the result was confusing and added to an already quite busy graph. We opted to leave the plots with the confidence intervals indicating uncertainty and clarifying in the legend that the values are meaningless and an artifact of the smoothing algorithm beyond the [0,1] interval.

-- In the legends of fig. S2 and S7, I would not call "circle" what is rather a dot or a point.

Fixed.

5- Typos:
-- Legend of fig S8: Tress -> Trees

Fixed.

-- Line 437: and -> an

Fixed.

Computer code is available, however it is not clear that the data numerical underlying most graphs is made available as a spreadsheet as recommended in the PLOS genetics policy

We have attached a spreadsheet with the numerical data underlying the figures in the manuscript (Numerical Data.xlsx).

Reviewer #3: In this article, the authors revisit a classic example of melanic colour variation in the chestnut-bellied flycatcher. They confirm that selection on two major pigmentation genes, *ASIP* and *MC1R*, is responsible for the independent emergence of melanic forms on Ugi and Santa Ana/Santa Catarina, respectively. They estimate the onset of selection and its strength, showing that melanism is likely older on Ugi than SA/SC, and under strong (s~0.01) selection. Their analyses take the demographic history into account. This is an excellent analysis using state of the art methods. The manuscript is clear and straightforward, and clearly shows the interest of this system for evolutionary biologists. There are many good ideas in terms of analyses, and the authors apply several descriptive (H1/H2, haplotype structure) and more complex (ARGs, machine learning) methods to interpret their data. Some results tend to be left aside (e.g. peaks of association on the Z chromosome, variant annotation near *ASIP*), as the authors mostly focus on candidate genes/variants that are already known in the system. This is not necessarily a problem in my opinion, but it should be made more explicit, and a brief plan for further analyses could be laid down at the end of the Discussion.

We thank the reviewer for the positive comments and helping improve the paper.

Major comments

Association analysis:

An intriguing finding is the peaks on the Z chromosome (mentioned L148, it would be good to highlight these peaks on Figure 2). Did the authors consider the sex of individuals when running their GWAS on Z scaffolds, since it could bias association tests? Assuming no bias due to heterogametic chromosomes, could this pattern reflect accelerated divergence on the Z due to lower effective population sizes, loci involved in reproductive isolation, or other loci involved in melanism? Are these variants in LD with variants near *ASIP*?

We now highlight the remaining peaks in Figure 2, have included a supplementary table (Table S1) showing the genes that are found within these peaks and a supplementary figure which shows that the variants in these peaks are in high LD (Figure S2). Our GWAS did not include the sex of individuals as a covariate, however most of the individuals in the comparison (and also in the Makira vs. SASC GWAS where we don’t find Z-linked hits) were male (~74%) making it unlikely that this is the reason why 3 of the 7 association peaks in the Makira vs. Ugi GWAS were on the Z sex chromosome. We have expanded the section on the additional association peaks, including those on the Z, mentioning the accelerated Z divergence and providing a reference.

Given the clear genetic structure, it may be interesting to use the demographic models to estimate the expected distribution of p-values for association for autosomes and sex-linked markers. The *p*-values seem quite low given the sample sizes, even for discrete traits with a simple genetic determinism. The authors mention Q-Q plots but I could not find them in the manuscript. Did the authors correct for genomic inflation (*λ*)?

Although we did not correct for genomic inflation we did correct for multiple testing. We applied a Bonferroni correction by correcting for the total number of comparisons conducted across both GWAS (i.e., a study-wide correction). We believe this is a conservative correction and note that the association peaks extend well beyond the significance cutoff.

Another intriguing aspect is the discrepancy between association and selection for *ASIP*. It could be indeed due to fixation/soft sweeps (as briefly mentioned L247-248). Authors run two GWAS analyses, grouping individuals from the main island with individuals from Ugi, then SA/SC. Given the strong genetic structure and demographic history, I am unsure that running two GWAS is the best option. A complementary method here could be BAYPASS (Gautier, 2015), to systematically compare allele frequencies across all populations/phenotypes at once in the analysis while correcting for relatedness between populations. You could then compare the association signal between the two analyses and their consistency. This could possibly eliminate some of the noise, or reveal signals that are less clear with the GWAS.

We have expanded and clarified the discussion on the signals of selection near *ASIP* and the possible reasons for this discrepancy. We now highlight the different statistics that support selection in the association peak on contig 947 and explain why we think we did not find strong signatures using SIA. We believe that the fixed loci in the Ugi population and the age of the mutations hinder our ability to detect selection when applying SIA. We therefore opted to expand our discussion rather than use and additional GWAS method.

Onset of selection:

I would recommend that the authors show the TMRCA for each population/phenotype (although this information is partially conveyed in the trees shown on Figure 3). This may help interpreting the lack of selection signal for associated variants near *ASIP*, and support their interpretation about an older, completed sweep. It may be interesting to examine the TMRCA of haplotypes found associated with melanism, to make the most of the information contained in the ARG.

We note that we provide the age of the mutations for *MC1R* and *ASIP* in the discussion (estimated through the midpoint age of the branch in which they first appeared; see methods).

In the case of *ASIP*, it seems likely that several cis-regulatory variants may play a role. If the authors intend to further investigate the system (and others) in a future work, I suggest Relate (Speidel, Forest, Shi, & Myers, 2019) to estimate genealogies along the genome, in combination with CLUES (<https://github.com/standard-aaron/clues>, (Stern, Speidel, Zaitlen, & Nielsen, 2021)) to test for polygenic selection.

CLUES in fact employs a similar approach to SIA that uses the full single-population genealogy to infer selection parameters. We discussed the differences in the approaches between SIA and CLUES and compared their performance in the SIA manuscript (<https://doi.org/10.1093/molbev/msab332>). The suggestion to test explicitly for polygenic selection using PALM (https://github.com/standard-aaron/palm), a derivative of CLUES, is a great idea and is something worth pursuing in a future work.

Functional variants (*ASIP*):
L159-160: It would be worth mentioning here the more distant peaks already (close to *EIF6* for example), as they may well correspond to important cis-regulatory variants. Studies in humans (among others) have shown signals of association quite far from *Agouti*. Moreover, given that differences in pigmentation are mostly found on the belly, it may be interesting to check position of GWAS hits relative to ventral promotor that have been described in, e.g., quail (Nadeau et al., 2008), if homolog sequences can be found.

We mention in the discussion the possibility of these additional mutations being cis-regulatory and note that the haplotypes shown in Figure S6 include those that are towards the end of the contig. We checked the Nadeau et al. (2008) quail paper which identified a deletion near *RALY* which places *ASIP* under the control of *RALY*’s promoter. The variants showing a high association in our study are in the opposite direction. Regardless, we agree that it would be interesting to better understand the contribution of cis-regulatory mutations to melanism in our study systems and plan on obtaining transcriptomic data in the future.

It might be worth to check for rearrangements (e.g. with DELLY? https://github.com/dellytools/delly) in the region. The presence of many associated SNPs at the end of the contig (i.e. near a region difficult to assemble) could be consistent with some sort of inversion. I understand the limitations of short reads for calling large structural variants, and leave it to the authors to decide whether this is feasible.

This is an interesting possibility, but as the reviewer mentioned we are limited in how we can explore it using short read data. Although we did assemble the reference genome using long reads in combination with short reads, all our resequencing data were obtained using short reads, making it hard to discover any population specific inversion. We checked the coverage in the candidate regions and did not find any patterns that stood out when comparing to the rest of the contig or to control contigs. Overall, it would be interesting to investigate the role of structural variants in the future using population-level long-read data.

**Minor comments**

L193: I would be a bit more cautious when discussing the efficiency of SIA. It is fine given that you have clear candidates for selection, but this would not be very satisfying for a naïve genome scan for selection. Confusion matrices suggest that the classifier cannot distinguish soft and hard sweeps that well, and the false positive rate for sweeps is around 8%. On Figure S9, what is the probability threshold to classify a window as neutral, hard sweep, soft sweep? How does the matrix change when changing the threshold for, say, p(neutral)?

Throughout the study, we took the class with the highest probability as inferred by SIA to be the predicted class. This is the strategy we used for creating the confusion matrices in Figure S10 as well as when applying the model to the data. We now clarify this in the Figure S10 caption and on lines 249-269, where we also explicitly state the false positive rate of this maximum class probability strategy estimated with the simulated data. To illustrate the behavior of the classifier with different probability threshold cutoff, we included ROC curves in panel B of Figure S10.

L208: Maybe show differentiation (e.g. F_ST_) between islands for SNPs that SIA identifies as under selection. Maybe show haplotype extension, for this region compared to *MC1R*. If it is indeed an ancient completed sweep, haplotypes should be shorter. The package rehh may be able to run quickly and works with unphased data: <https://cran.r-project.org/web/packages/rehh/vignettes/rehh.html>

We have expanded the discussion on the sweeps in the *ASIP* region where it’s likely that the coding mutation initially swept to fixation, but that there has been subsequent selection on other perhaps cis-regulatory regions. Consequently it’s hard to have a prediction on the haplotype length if there have been multiple selection events. The association peak involving *MC1R* is ~150 kb long, while the one involving *ASIP* is in fact much longer (~650 kb long). This can be seen in Figure 2 in combination with the genotype plots in Figures S4 and S6. Figure S3A also shows the generally higher differentiation within the association peak (where SIA identified positions under selection) and outside.

L336: Explain why you mask the genome before gene annotation (to avoid annotating TEs as genes I assume). This can be useful for readers not familiar with genome assembly.

Agreed. We now mention this on lines 462-464.

L438: Mutation rate seems a bit low (10^-9^) compared to other rates that are closer to 2.10^-9^/year. Briefly mention how this can change your demographic estimates.

It is true that we use an approximate mutation rate estimated for birds (reference 65) and that our absolute estimates of population size and divergence time (though not migration rates) will be impacted by this assumption. However, we try to focus our interpretation on relative comparisons which are independent of the assumed mutation rate. We have clarified this point in the manuscript (Lines 597-601).

I hope you find these comments constructive. Congratulations for this nice work.

Yann Bourgeois

Thank you very much for your time and effort to improve the manuscript

**References**

Gautier, M. (2015). Genome-Wide Scan for Adaptive Divergence and Association with Population-Specific Covariates. *Genetics*, *201*(September), 1555–1579. doi: doi:0.1534/genetics.115.181453

Nadeau, N. J., Minvielle, F., Ito, S., Inoue-Murayama, M., Gourichon, D., Follett, S. a, … Mundy, N. I. (2008). Characterization of Japanese quail yellow as a genomic deletion upstream of the avian homolog of the mammalian ASIP (agouti) gene. *Genetics*, *178*(2), 777–786. doi: 10.1534/genetics.107.077073

Speidel, L., Forest, M., Shi, S., & Myers, S. R. (2019). A method for genome-wide genealogy estimation for thousands of samples. *Nature Genetics*, *51*(9), 1321–1329. doi: 10.1038/s41588-019-0484-x

Stern, A. J., Speidel, L., Zaitlen, N. A., & Nielsen, R. (2021). Disentangling selection on genetically correlated polygenic traits via whole-genome genealogies. *American Journal of Human Genetics*, *108*(2), 219–239. doi: 10.1016/j.ajhg.2020.12.005
