# Supplementary material for: Selective sweeps on different pigmentation genes mediate convergent evolution of island melanism in two incipient bird species
Source: PLoS Genet. 2022 Nov 1;18(11):e1010474. doi: 10.1371/journal.pgen.1010474 (PMC9624418; doi:10.1371/journal.pgen.1010474)
Supplement: Attachment — Submitted filename: R2_Plos_Genetics_V2.docx [file pgen.1010474.s017.docx]

October 3^rd^, 2022

Dr. Nicolas Bierne

Academic Editor

PLOS Genetics

Dear Dr. Bierne,

Thank you for your letter from September 29^th^ regarding our manuscript PGENETICS-D-22-00667. We have clarified the discussion on the complex signals of selection in the region surrounding ASIP and modified the title of the manuscript following your suggestion. We also provide details on our interpretation of the data below.

We thank you for the opportunity to revise our manuscript, and for the helpful suggestions.

Sincerely,

Leonardo Campagna on behalf of all co-authors.

Thank you for your work of revision. I think you have accurately addressed referees’ concerns and that your ms is near ready for definitive acceptance.

We appreciate the effort made by the reviewers’ and yourself to improve our manuscript.

However I would like to give you a last opportunity to improve the clarity of the explanations about the ASIP contig sweep(s). Indeed I am not entirely satisfied with the “in and/or around” argumentation. Either it’s in, or it’s around, but “in and around” is not clear to me.

We no longer use this terminology for the *ASIP* contig. The case for *MC1R* is different as the variants within and around *MC1R* are found on the same haplotype, and therefore selection occurred on all of these variants.

You have a GWAS peak, a differentiation peak, an amino acid change (Ile55Thr), and a shallow genealogy (Fig. S6, S8) at ASIP (position ~6.6Mb), and a second GWAS peak, differentiation peak, soft-sweep like genealogy at the 5’ side of MMP24 (position ~7Mb). The interpretation of these two regions would need to be better articulated, not just mixed in a single blurred “in or around” argumentation. To say it more explicitly, what if the 7Mb region was the soft shoulder of a harder sweep (Schrider et al. 2015 [doi.org/10.1534/genetics.115.174912](http://doi.org/10.1534/genetics.115.174912))?

Thank you for this suggestion, we have attempted to clarify our interpretation in the discussion. First, it is possible that the signatures of selection between 6.9-7 Mb are related to cis-regulatory variants as suggested by Reviewer 3. Without speculating too much, these mutations could modify *ASIP*’s direct effects on coloration or pleiotropic effects unrelated to color (e.g., stress response, metabolism and food intake; reviewed by Ducrest et al. 2008, DOI: 10.1016/j.tree.2008.06.001). A second possibility could be that this downstream position involves selection that is independent of *ASIP* and coloration, as may be the case for the remaining association peaks. Indeed, the Ugi and Makira populations show other phenotypic differences like body size which covaries with melanism. Finally, we also discuss your suggestion on the soft shoulder of a hard sweep. Although this is possible, we think it is less likely as we tested this possibility in a similar framework applied to a different dataset, and that type of misclassification was not common (<https://doi.org/10.1073/pnas.2015987117>; SI page 21 and Table S7). In that paper the classifier had been trained using summary statistics, which is slightly different from the more powerful ARG approach used by SIA. We discuss these possibilities in lines 336 to 341 of the revised manuscript.

How is the age of the Ile55Thr substitution dated? If recombinants are included in the window, the age can be much overestimated.

We dated the origin of the mutation by using the ARG and estimating the midpoint age of the branch in which it first appeared. It shouldn’t have that issue of overestimation.

What’s the idea in the consecutive sweeps scenario, that the second one makes the plumage blacker? Please try to rework on clarifying the explanations.

It is possible that the second mutation fine-tunes the expression of *ASIP* (e.g., in coloration or pleiotropic contexts; see discussion mentioned above) now that *ASIP* carries a coding mutation. A type of compensatory mutation.

I still think the title is misleading. You’d rather say “selective sweeps on independent/different/alternative pigmentation genes” than “independent selective sweeps on pigmentation genes”.

We have followed this suggestion and modified the title accordingly.

Finally, we realized we were using both Chestnut-bellied Flycatcher and Chestnut-bellied Monarch as the common English name of the study species. Although both names are used in the literature, the latter is the one preferred by taxonomic authorities. We therefore changed all references to the study species to Chestnut-bellied Monarch.
